# Tumor cell sensitivity to vemurafenib can be predicted from protein expression in a *BRAF*-V600E basket trial setting

**DOI:** 10.1186/s12885-019-6175-2

**Published:** 2019-10-31

**Authors:** Molly J. Carroll, Carl R. Parent, David Page, Pamela K. Kreeger

**Affiliations:** 1 Department of Biomedical Engineering, University of Wisconsin-Madison 1111 Highland Ave, WIMR 4553, Madison, WI 53705 USA; 2 Department of Biostatistics and Bioinformatics, Duke University, Box 2721, Durham, NC 27710 USA; 3 University of Wisconsin Carbone Cancer Center, University of Wisconsin School of Medicine and Public Health, Madison, WI USA; 4 Department of Obstetrics and Gynecology, University of Wisconsin School of Medicine and Public Health, Madison, WI USA; 5 Department of Cell and Regenerative Biology, University of Wisconsin School of Medicine and Public Health, Madison, WI USA

**Keywords:** Reverse phase protein array, Orthogonal partials least squares, Protein activity, Targeted therapies, BRAF inhibitor

## Abstract

**Background:**

Genetics-based basket trials have emerged to test targeted therapeutics across multiple cancer types. However, while vemurafenib is FDA-approved for *BRAF*-V600E melanomas, the non-melanoma basket trial was unsuccessful, suggesting mutation status is insufficient to predict response. We hypothesized that proteomic data would complement mutation status to identify vemurafenib-sensitive tumors and effective co-treatments for *BRAF*-V600E tumors with inherent resistance.

**Methods:**

Reverse Phase Proteomic Array (RPPA, MD Anderson Cell Lines Project), RNAseq (Cancer Cell Line Encyclopedia) and vemurafenib sensitivity (Cancer Therapeutic Response Portal) data for *BRAF*-V600E cancer cell lines were curated. Linear and nonlinear regression models using RPPA protein or RNAseq were evaluated and compared based on their ability to predict *BRAF*-V600E cell line sensitivity (area under the dose response curve). Accuracies of all models were evaluated using hold-out testing. CausalPath software was used to identify protein-protein interaction networks that could explain differential protein expression in resistant cells. Human examination of features employed by the model, the identified protein interaction networks, and model simulation suggested anti-ErbB co-therapy would counter intrinsic resistance to vemurafenib. To validate this potential co-therapy, cell lines were treated with vemurafenib and dacomitinib (a pan-ErbB inhibitor) and the number of viable cells was measured.

**Results:**

Orthogonal partial least squares (O-PLS) predicted vemurafenib sensitivity with greater accuracy in both melanoma and non-melanoma *BRAF*-V600E cell lines than other leading machine learning methods, specifically Random Forests, Support Vector Regression (linear and quadratic kernels) and LASSO-penalized regression. Additionally, use of transcriptomic in place of proteomic data weakened model performance. Model analysis revealed that resistant lines had elevated expression and activation of ErbB receptors, suggesting ErbB inhibition could improve vemurafenib response. As predicted, experimental evaluation of vemurafenib plus dacomitinb demonstrated improved efficacy relative to monotherapies.

Conclusions: Combined, our results support that inclusion of proteomics can predict drug response and identify co-therapies in a basket setting.

## Background

In recent decades, there has been a shift to add targeted therapeutics (e.g.*,* Herceptin) to standard cancer treatment approaches such as surgery, chemotherapy, and radiation. This is due, in part, to the emergence of large-scale DNA sequence analysis that has identified actionable genetic mutations across multiple tumor types [1, 2]. For example, mutations in the serine-threonine protein kinase *BRAF* are present in up to 15% of all cancers [3], with an increased incidence of up to 70% in melanoma [4]. In 2011, a Phase III clinical trial for vemurafenib was conducted in *BRAF*-V600E melanoma patients with metastatic disease [5]. Based on the significant improvements observed for both progression-free and overall survival, vemurafenib was subsequently FDA-approved for first-line treatment of metastatic, non-resectable melanoma.

However, conducting a clinical trial for a targeted therapeutic can be challenging due to slow patient accrual, particularly for tumor types that harbor the mutation at a low frequency [2]. To combat this challenge, basket trials have emerged as a method where multiple tumor types harboring a common mutation are entered collectively into a single clinical trial [6]. Unfortunately, results of the basket clinical trial of vemurafenib for non-melanoma tumors with the *BRAF*-V600E mutation indicated that other cancers, including colorectal, lung, and ovarian responded poorly to vemurafenib monotherapy [7]. However, some patients exhibited a partial response or achieved stable disease, suggesting that information beyond the presence of a genetic mutation might identify potential responders in a basket setting. Additionally, a subset of colorectal patients achieved a partial response when combined with cetuximab, suggesting that the effects of vemurafenib are subject to the larger cellular network context.

To better identify patient cohorts that will respond to targeted therapeutics, precision medicine approaches have begun to use machine learning algorithms to find associations between drug sensitivity and “omic” data such as gene expression and mutational status. Consistent with the basket trial result for melanoma, one such study found that mutation status was an imperfect predictor across multiple cancer types and drugs [8]. While most prior studies have examined transcriptomic data to predict drug sensitivity [9], a few studies have examined protein expression and activation to predict response to therapies [10, 11]. A recent study showed that models built with protein expression were better able to predict sensitivity to inhibitors of the ErbB family of receptors compared to gene expression, suggesting protein expression may be more informative [12].

However, the studies performed by Li et al. analyzed cell lines independent of their genomic status. This may limit the translational potential of this approach as mutational status is a primary criteria for many targeted therapy trials due to the relative ease of developing companion diagnostics for single mutations. We hypothesize that in a basket setting, the addition of protein expression and activity will provide superior predictive power compared to mutation status alone and will lead to identification of co-therapies to improve responses for cells with inherent resistance. To address this hypothesis, we built and compared multiple machine learning models from a publicly available RPPA dataset for 26 *BRAF-*V600E pan-cancer cell lines and identified protein signatures predictive of sensitivity to the FDA-approved BRAF inhibitor vemurafenib. From these signatures, potential co-therapies were identified and their respective impacts on vemurafenib efficacy were tested.

## Materials and methods

### Cell lines and reagents

Unless otherwise stated, all reagents were purchased from ThermoFisher (Waltham, MA). Cancer Cell Line Encyclopedia lines A375, LS411N, and MDAMB361 were purchased from American Type Culture Collection (ATCC; Rockville, MD). Cells were maintained at 37 °C in a humidified 5% CO_2_ atmosphere. A375 and LS411N were cultured in RPMI 1640 supplemented with 1% penicillin/streptomycin and 10% heat-inactivated fetal bovine serum. MDA-MB-361 were cultured in RPMI 1640 supplemented with 1% penicillin/streptomycin, 15% heat-inactivated fetal bovine serum, and 0.023 IU/mL insulin (Sigma; St. Louis, MO).

### Matching CCLE, RPPA, and CTRP cell data

*BRAF-*V600E mutational status of cancer cell lines was obtained through the CCLE portal (https://portals.broadinstitute.org/ccle, Broad Institute; Cambridge, MA). The RPPA data for the 26 *BRAF* mutated cancer cell lines (Additional file 1: Table S1) was generated at the MD Anderson Cancer Center as part of the MD Anderson Cancer Cell Line Project (MCLP, https://tcpaportal.org/mclp) [12]. Of the reported 474 proteins in the level 4 data, a threshold was set that for inclusion a protein must be detected in at least 25% of the selected cell lines, resulting in 232 included in the analysis. Gene-centric RMA-normalized mRNA expression data was retrieved from CCLE portal. Data on vemurafenib sensitivity was collected as part of the Cancer Therapeutics Response Portal (CTRP; Broad Institute) and normalized area-under-IC50 curve data (IC_50_AUC) was procured from the Quantitative Analysis of Pharmacogenomics in Cancer (QAPC, http://tanlab.ucdenver.edu/QAPC/) [13].

### Regression algorithms to predict vemurafenib sensitivity

Regression of vemurafenib IC_50_AUC with RPPA protein expression was analyzed by Support Vector Regression with linear and quadratic polynomial kernels (SMOreg, WEKA [14]), cross-validated least absolute shrinkage and selection operator (LASSOCV, Python; Wilmington, DE), cross-validated Random Forest (RF, randomly seeded 5 times, WEKA), and O-PLS (SimcaP+ v.12.0.1, Umetrics; San Jose, CA) with mean-centered and variance-scaled data. Models were trained on a set of 20 cell lines and tested on a set of 6 cell lines (Additional file 2: Table S2). Root mean squared error of IC_50_AUC in the test set was used to compare across regression models using the following formula:

1 $$ {RMSE}_{pred}=\sqrt{\frac{\sum \limits_{i=1}^n{\left({\hat{y}}_i-{y}_i\right)}^2}{n}} $$

In O-PLS model, R^2^Y, the coefficient of determination for predicted behavior Y, describes how well the model fits the predicted behavior, while Q^2^Y measures the predictive value of the model based upon 7-fold cross validation. Predictive and orthogonal components were defined sequentially, and if Q^2^Y increased significantly (> 0.05) with the addition of the new component, that component was retained, and the algorithm continued until Q^2^Y no longer significantly increased. The variable importance of projection (VIP) score summarizes the overall contribution of each protein’s measurement to the O-PLS model, and the VIP score for variable *j* is defined via the following equation:

2 $$ {VIP}_j=\sqrt{\frac{p}{\sum \limits_{m=1}^M SS\left({b}_m\bullet {t}_m\right)}\bullet \sum \limits_{m=1}^M{w}_{mj}^2\bullet SS\left({b}_m\bullet {t}_m\right)} $$

where *p* is the total number of variables, *M* is the number of principal components, *w*_*mj*_ is the weight for the *j*-th variable in the *m*-the principal component and *SS (b*_*m*_*∙t*_*m*_*)* is the percent variance in *y* explained by the *m*-th principal component. Proteins whose VIP score is greater than 1 are considered important towards the predictive power of the model.

For a receptor-only built O-PLS model, expression of AR, CMET, CMET-Y1235, EGFR, EGFR-Y1068, EGFR-Y1173, ERα, ERα-S118, HER2, HER2-Y1248, HER3, HER3-Y1289, IGFRB, PDGFRB, PR, and VEGFR2 were used to predict vemurafenib IC_50_ AUC, using all 26 cell lines for training. To simulate pan-ErbB inhibition for MDA-MB-361, LS411N, and A375, the RPPA values for EGFR, HER2, and HER3 phosphorylated receptors were set to each protein’s minimum value in the original data set.

### Heatmaps and clustering

Mean-centered and variance scaled RPPA data for training and testing set cell lines were hierarchically clustered (1-Pearson) with publicly available Morpheus software (https://software.broadinstitute.org/morpheus, Broad Institute). Resulting heatmap plots were created in GraphPad Prism software (La Jolla, California).

### CausalPath analysis of resistant cell lines

CausalPath software [15] was used to identify networks of proteins from the RPPA data set that were significantly enriched in the resistant cell lines (IC_50_ AUC < 0.2) compared to the sensitive cell lines. For analysis of predictive protein interactions, proteins with a VIP > 1 were examined (87 of the original 232 proteins met this criteria), and significant change in the mean expression of each protein/phosphorylated protein between the two groups was determined with 10,000 permutations and a FDR of 0.2 for total and phosphorylated proteins. This relaxed discovery rate is consistent with prior use of this algorithm with a constrained subset of proteins [15].

### In vitro testing of co-therapeutics

A375, LS411N, and MDAMB361 were seeded at 3000 cells/cm^2^, 5000 cells/cm^2^, and 10,000 cells/cm^2^ respectively in duplicate in 96 well opaque, white assay plates for 24 h. Vemurafenib (Santa Cruz Biotechnology; Dallas, TX), dacomitinib, or a 1:2 dual treatment of vemurafenib:dacomitinib were tested using 2-fold concentration ranges (highest concentration of 33 μM and 66 μM respectively) for 72 h. ATP levels were measured using CellTiter-Glo (Promega; Madison, WI) to assess cell viability. ATP levels were simultaneously measured in cells treated with vehicle (0.2% DMSO) cells, and all values were corrected by subtraction of measurements from blank wells. The ATP level of vehicle-treated cells was set as A_min_ and percent inhibition was calculated using the following formula:

3 $$ y=\frac{\left({A}_{min}-x\right)}{A_{min}}\times 100 $$

GraphPad was used to calculate nonlinear log (inhibitor) fit of each dose response curve using the following formula:

4 $$ y=\frac{100}{{\left(1+\frac{IC_{50}}{x}\right)}^{Hill}} $$

where the Hill coefficient is the Hill slope of the best fit line calculated by GraphPad.

Loewes additive model [16] was used to determine synergy between monotherapy and dual therapy treatments using the following formula:

5 $$ \frac{x^1}{X_{LOEWE}^1}+\frac{x^2}{X_{LOEWE}^2} $$

where x^1^, x^2^ represent the dual therapy IC_50_ concentrations for each drug, and X^1^_LOEWE_, X^2^_LOEWE_ represent the monotherapy IC_50_ for each drug. Model values less than 1 indicate synergy.

### Statistical analysis

To compare the different machine learning models, each model was evaluated on all 26 cell lines using leave one out cross validation. Errors for each cell line prediction were calculated, and models were evaluated on the number of cell lines for which they had the smallest error in comparison with O-PLS. A binomial t-test was performed in Prism for each model against O-PLS.

## Results

### Tumors exhibit heterogeneous protein expression and sensitivity to vemurafenib

**Fig. 1:**
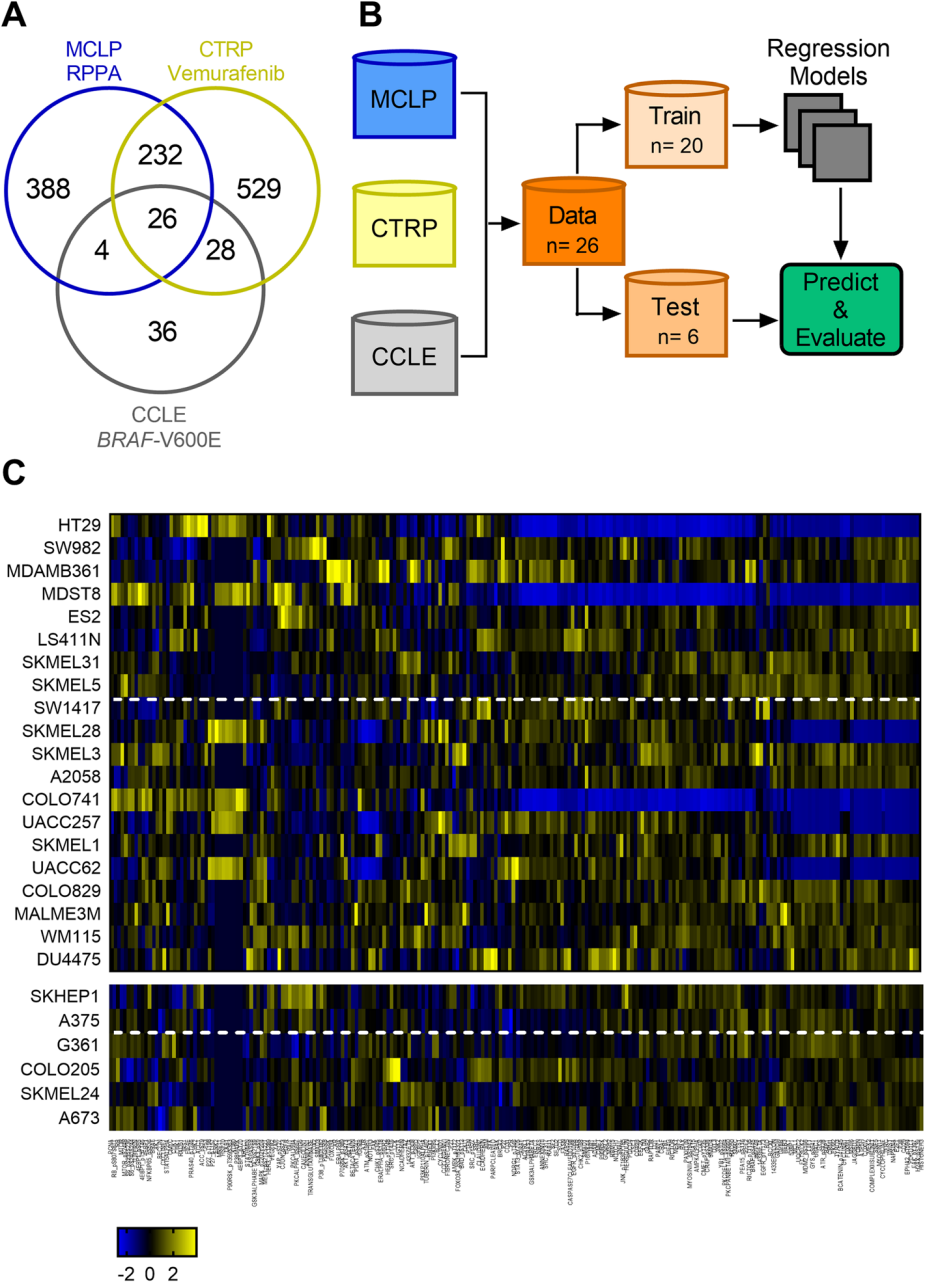
Overview of dataset curation. (**a**) Intersection of number of cell lines represented in the MCLP RPPA level 4 dataset, CTRP vemurafenib response dataset, and CCLE database of *BRAF*-V600E mutated cells. (**b**) Pipeline of data curation and evaluation of machine learning models to predict vemurafenib response in *BRAF*-V600E cell lines. (**c**) Heatmap illustrating z-score normalized expression of 232 proteins used in model evaluation. Top heatmap indicates training set and bottom indicates testing set of cell lines in order of increasing IC_50_ AUC, with cell lines above the dotted line having IC_50_ AUC < 0.2

To examine the ability of protein expression and activity to predict response in *BRAF*-V600E tumor cells to the BRAF inhibitor vemurafenib, appropriate cell line models were explored. Of the cell lines characterized by the Cancer Cell Line Encyclopedia (CCLE) that possess a *BRAF-*V600E mutation (*n* = 94), and the Reverse Phase Protein Array (RPPA) data available from the MD Anderson Cell Line Project (MCLP, *n* = 650), 26 overlapped and had data pertaining to vemurafenib sensitivity in the Cancer Therapeutic Response Portal (CTRP) (Fig. 1 a, Additional file 1: Table S1). While many studies have predicted the dose of a drug that inhibits tumors by 50% (IC_50_), analysis of IC_50_ doses of vemurafenib in these 26 cell lines showed that many exceeded the maximal dose tested in the CTRP database [13, 17]. Therefore, the normalized area under the dose response inhibition curve (IC_50_ AUC) was used as a measure of vemurafenib sensitivity. This response metric has been used in other pharmacogenomic studies to better capture sensitivity of cells to a drug, either using AUC < 0.2 as a classifier of resistant cell lines, or predicting sensitivity as a continuous response (0 < AUC < 1) [18]. Analysis of the 26 cell lines showed that, like patient responses to vemurafenib [5, 7], most non-melanoma cell lines were resistant to vemurafenib (AUC < 0.2, *n* = 7/11), while most melanoma cell lines were sensitive to vemurafenib (AUC > 0.2, *n* = 12/15, Additional file 1: Table S1). However, because the range captured in the response to vemurafenib is broad (10^− 4^ - 0.97), we aimed to predict the continuous response to vemurafenib, rather than classify resistant and sensitive cells alone.

### Orthogonal partial least squares model outperforms other regression models to predict vemurafenib sensitivity

Since the goal was to predict the continuous IC_50_ AUC in *BRAF* mutated cell lines based on their RPPA protein expression data, we compared various types of regression models to determine the model that performed with the highest accuracy. Regression models, such as support vector regression (SVR) with linear kernels, orthogonal partial least squares regression (O-PLS), and LASSO-penalized linear regression, utilize linear relationships between the protein expression and vemurafenib sensitivity for prediction. One limitation of our data set is the relatively low number of cell lines (observations, *n* = 26) relative to RPPA proteins (variables, *n* = 232); given a data set with more variables than observations, over-fitting of the training data is always a concern. O-PLS addresses this issue by reducing the dimension to predictive and orthogonal principal components that represent linear combinations of the original protein expression cohort [19], while LASSO-penalized regression instead addresses the same issue by introducing an *L*_*1*_ regularization term that penalizes non-zero weights given to proteins in the model [20]. While these two model types are restricted to linear relationships, Random Forests (with regression trees) and SVRs with non-linear kernels possess the ability to find non-linear interactions between proteins to predict vemurafenib sensitivity. Random Forests address overfitting via the use of an ensemble approach, making predictions by an unweighted vote among multiple trees, while SVRs at least partially address overfitting by not counting training set errors smaller than a threshold ε, i.e.*,* not penalizing predictions that are within an “ε-tube” around the correct value [21, 22].

**Fig. 2:**
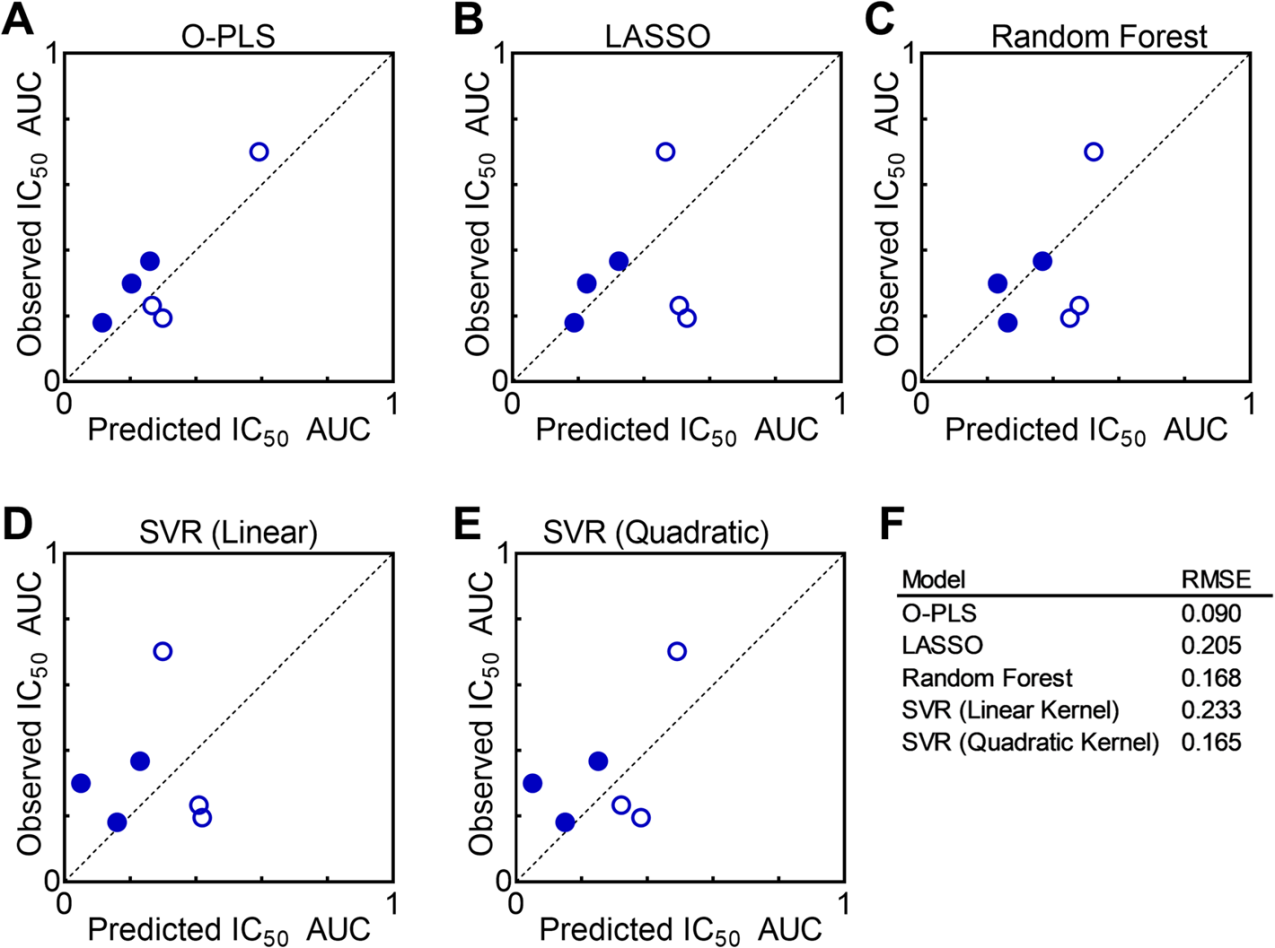
Comparison of machine learning algorithm predictions of vemurafenib sensitivity. Comparison of prediction performance on the testing set of cell lines for (**a**) O-PLS, (**b**) LASSO, (**c**) Random Forest, (**d**) SVR with linear kernel and (**e**) SVR with quadratic kernel. Open symbols indicate melanoma cell lines, closed symbols indicate non-melanoma cell lines. (**f**) RMSE for prediction set of each model

To evaluate SVRs (using linear and quadratic kernels), LASSO, Random Forest, and O-PLS algorithms, the original set of 26 cell lines was split into a training set of 20 and testing set of 6 cell lines (Fig. 1b,c, Additional file 1: Table S1). To represent the full variability in the data set, the training/testing split was not entirely random, but rather ensured that each set contained at least one each of: a melanoma cell line with IC_50_ AUC > 0.2, a melanoma cell line with IC_50_ AUC < 0.2, a non-melanoma cell line with IC_50_ AUC > 0.2, and a non-melanoma cell line with IC_50_ AUC < 0.2. Figure 2 and Additional file 2: Table S2 summarize the performance of these five algorithms to predict vemurafenib sensitivity from the 232 proteins in the RPPA dataset. Overall, O-PLS was the most accurate in predicting the IC_50_ AUC metric across the 6 validation set cell lines (RMSE = 0.09; binomial test, Additional file 3: Table S3), and performed well predicting both non-melanoma and melanoma cell lines (Fig. 2a,f). The LASSO and Random Forest models (Fig. 2b,c,f) performed second best in terms of RMSE across the 6 cell lines; however, these model forms appeared to overestimate IC_50_ AUC for melanoma cell lines and underestimate IC_50_ AUC for non-melanoma cell lines, resulting in larger prediction errors for melanoma cell lines compared to non-melanoma (Additional file 3: Table S3). The SVR model with a linear kernel had the highest error for the prediction set (RMSE = 0.233), and while use of a quadratic kernel decreased the error, interpretability of this model was decreased due to the non-linear interactions (Fig. 2d-f, Additional file 3: Table S3). Based on our goals of pan-cancer accuracy and ease in model interpretation, we selected to analyze the O-PLS model in greater depth.

### O-PLS identifies unique protein signatures that correlate with vemurafenib sensitivity

**Fig. 3:**
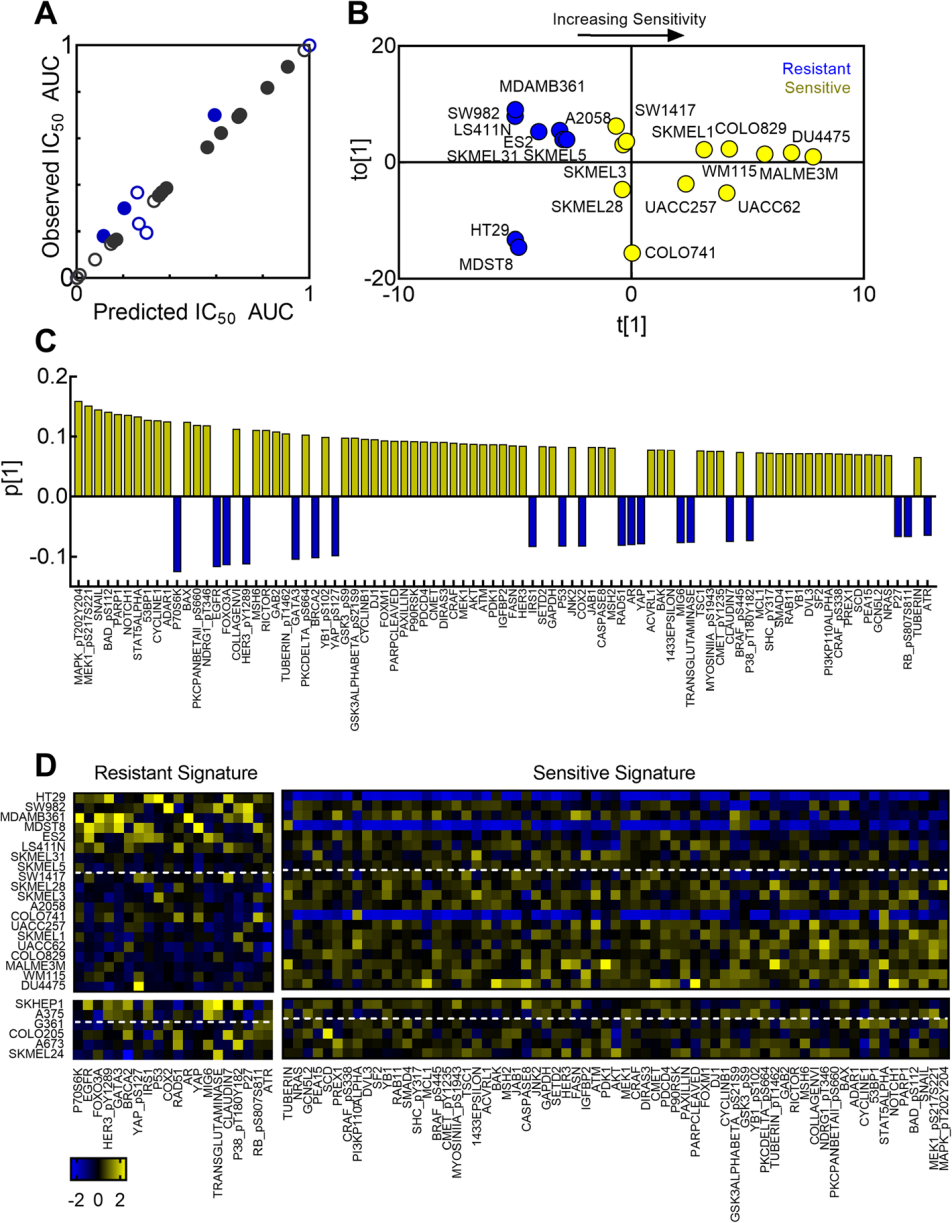
O-PLS prediction of vemurafenib sensitivity from RPPA dataset. (a) Comparison of observed and predicted IC_50_ AUC values in training (7-fold cross validation) and testing set cell lines. Open symbols indicate melanoma cell lines, closed symbols indicate non-melanoma cell lines. (b) Scores plot of O-PLS model showing projection of training cells along first component t[1] and first orthogonal component to [1]. (c) Weights of proteins (VIP score > 1) along the predictive component. (d) Heatmap of z-score normalized proteins (VIP score > 1) whose weights correlate with resistant (left) and sensitive cell lines (right). Top heatmap indicates training set and bottom indicates testing set of cell lines in order of increasing IC_50_ AUC, with cell lines above the dotted line having IC_50_ AUC < 0.2

The O-PLS model accurately captured the high variance in vemurafenib sensitivity (R^2^Y = 0.99), had the most accurate prediction in the single train-test split previously described, and maintained reasonable prediction accuracy during cross validation (Q^2^Y = 0.4, Fig. 3a). The cell lines projected along the first component t[1] according to increasing IC_50_ AUC, while they projected along the orthogonal component t_o_[1] according to tumor type of the cell line (Fig. 3b). For instance, while the two triple negative breast cancer cell lines MDA-MB-361 and DU-4475 have differing vemurafenib sensitivity, they project within the same orthogonal principal component space (Fig. 3b). Further analysis of the first and orthogonal component showed that the first component captured a lower percentage of the variance in the protein expression compared to the orthogonal component (R^2^X_pred_ = 0.08, R^2^X_orthog_ = 0.36). Additionally, removal of the orthogonal component to produce an O-PLS model using only the first component reduced the predictive power of the model (Q^2^Y = 0.0842). These results suggest that the improved prediction success of O-PLS may result from its use of orthogonal components, which here identify and distinguish protein expression patterns that correlate to tumor type independent of protein patterns that correlate to vemurafenib-sensitivity.

Of the 232 proteins from the RPPA dataset used in this model, 87 had VIP scores greater than 1, and were thus the most important proteins for the prediction of this model. Figure 3c illustrates these proteins with respect to their weights along p[1]. A small subset of proteins and phosphorylated forms of proteins correlated with projection along the negative space of p[1], suggesting that high levels of these proteins were associated with intrinsic resistance to vemurafenib (Fig. 3c, blue). Further inspection of the expression of these proteins in both the training and testing set showed that, on average, these proteins were more highly expressed in resistant cell lines (IC_50_ AUC < 0.2, Fig. 3d). Included in this signature were both EGFR and a phosphorylated form of HER3 (HER3 Y1289), as well as downstream signaling proteins in the AKT pathway, such as P70S6K, suggesting that expression and activity of this family of receptors and downstream pathways correlate with increased vemurafenib resistance. Conversely, the protein signature that correlated with increased sensitivity to vemurafenib included proteins in the MAPK pathway such as NRAS, BRAF S445, MEK S217/S221, MAPK T202/Y204 (Fig. 3c yellow bars, Fig. 3d). This suggests that even among cell lines that universally possess a constitutively activating mutation in *BRAF*, increased activation of this pathway correlated with increased sensitivity.

### Protein expression and activity outperform gene expression for predicting vemurafenib sensitivity

**Fig. 4:**
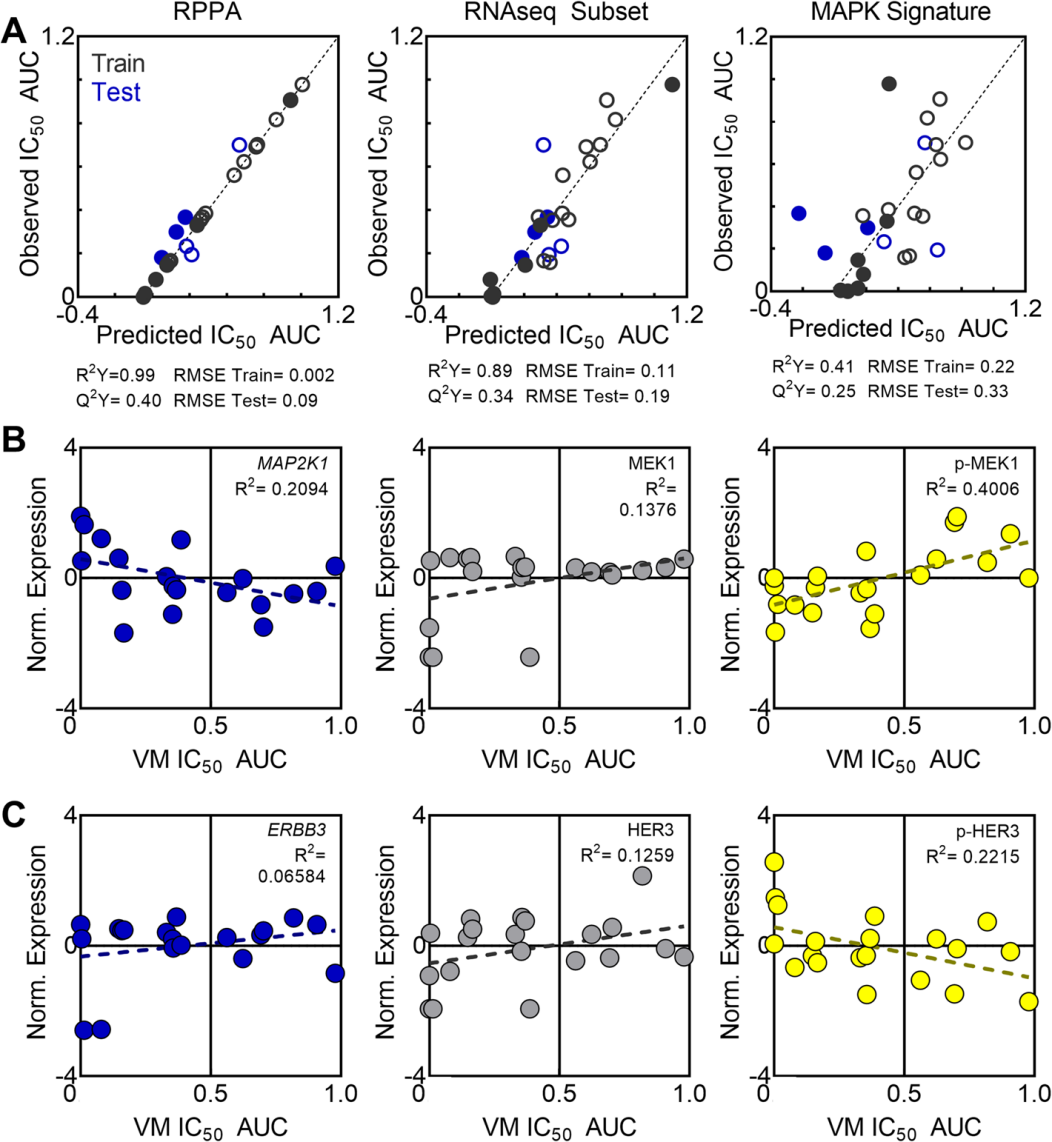
O-PLS prediction of vemurafenib sensitivity from different data forms. (**a**) Comparison of O-PLS model performances for training (7-fold cross validation, grey) and testing sets of cell lines (blue). Models were built on the RPPA dataset (RPPA), gene expression corresponding to RPPA proteins (RNAseq Subset), or gene expression of the MAPK signature (MAPK signature). Open symbols indicate melanoma cell lines, closed symbols indicate non-melanoma cell lines. (**b**, **c**) Comparison of univarate correlations of z-score normalized gene expression (blue), total protein expression (grey) and phospho-protein expression (yellow) of MEK1 (**b**) and HER3 (**c**) with IC_50_ AUC

While the O-PLS model utilized a pharmaco-proteomics approach, others have used transcriptomic data to predict therapeutic responses in tumor cell lines [18, 23]. To examine the relative strength of proteomic vs. transcriptomic data, we revised the model to predict vemurafenib sensitivity in *BRAF* mutated cell lines from RNAseq data curated by the CCLE. In the first RNAseq model comparison, we predicted vemurafenib sensitivity from genes in the RNAseq dataset that corresponded to proteins represented in the 232 protein RPPA data set (RNAseq Subset). In comparison to the O-PLS model built on RPPA protein expression (Fig. 3a, reproduced in 4A, left for direct comparison), the RNAseq Subset model was less able to capture the variance in sensitivity (R^2^Y = 0.89 vs. 0.99) and was less predictive (Q^2^Y = 0.34 vs. 0.40). Additionally, this change resulted in an increased RMSE during model evaluation on the training set using 7-fold cross validation, as well as an overestimation of melanoma cell lines in the testing set (Fig. 4a middle, Additional file 4: Table S4). Previously, a MAPK pathway activity score was developed from the expression of 10 genes to identify cell line and patient response to variety of MAPK pathway inhibitors, including vemurafenib [24]. While developed from data from patients both with and without the *BRAF-*V600E mutation, this signature performed best for *BRAF-*V600E melanoma patients. To investigate this *MAPK* signature in our basket setting, a model was built to predict vemurafenib sensitivity from RNAseq expression of the 10 genes in the signature. Evaluation of this model showed that variance captured in vemurafenib sensitivity was the lowest of these three models (R^2^Y = 0.53). Additionally, this model iteration showed the lowest predictive ability between the three O-PLS models tested (Q^2^Y = 0.31) and the highest error in the training set (7-fold cross validation) and the test set of cell lines, particularly in non-melanoma cell lines (Fig. 4 a right, Additional file 2:Table S2 and Additional file 4:Table S4). To further investigate why protein expression and activity may better predict sensitivity to vemurafenib compared to RNAseq data, we calculated univariate correlations of phosphoprotein expression for predictive phosphoproteins (VIP score > 1) in the RPPA, gene expression and/or total protein expression with vemurafenib sensitivity (IC_50_ AUC, Fig. 4b,c, Additional file 5: Table S5). Not surprisingly, all univariate relationships were weaker than the multivariate O-PLS model for either RPPA or RNAseq. Of the phosphoproteins with VIP score > 1, 10/13 had higher correlation coefficients (R^2^) than their total protein expression, and 14/18 had higher correlation than the gene expression, including p-MEK1 (R^2^ = 0.4006) and p-HER3 (R^2^ = 0.2215). Notedly, some gene/protein pairs such as *MAP2K1*/MEK1 had discordant trends in the correlation with sensitivity (Fig. 4b). Alternatively, for some gene/protein pairs there was a similar trend, but a discordance was instead observed at the phosphoprotein level (*ERBB3*/HER3/p-HER3, Fig. 4c). These results suggest that protein expression and activity may be a more direct readout of pathway activity compared to gene expression in cells. To explore this further, O-PLS models were built using either expression of total proteins (*n* = 173 variables) or phosphorylated proteins (*n* = 59 variables) represented in the RPPA dataset. The O-PLS model built from total protein expression maintained the high variance in IC_50_ AUC captured from the original full RPPA (*n* = 232 variables) O-PLS model (R^2^Y = 0.99 for both) but had lower predictive ability (Q^2^Y = 0.37 vs. Q^2^Y = 0.40). Additionally, the total protein O-PLS model had higher error in prediction for the held aside test set (RMSE = 0.11 vs. RMSE = 0.09, Additional file 6: Table S6 and Additional file 8: Fig. S1A). Further inspection found the O-PLS model built from total protein expression made greater prediction errors on non-melanoma cell lines in the held aside test set (Additional file 6: Table S6). In the O-PLS model built on phosphoproteins, the variance captured in IC_50_ AUC, the predictive ability of the model, and the accuracy in the held aside test set suffered (R^2^Y = 0.43, Q^2^Y = 0.09, RMSE = 0.19). However, this phosphoprotein-built O-PLS favored more accurate prediction of non-melanoma cell lines (Additional file 8: Fig. S1B, Additional file 6: Table S6). Overall, the correlation analysis and O-PLS model comparisons showed that vemurafenib sensitivity was more accurately predicted from proteomic data than genomic data, and that incorporation of protein phosphorylation may be important to capturing vemurafenib sensitivity across a wide range of tumor types.

### ErbB receptor activation and downstream PI3K signaling is increased in vemurafenib-resistant cell lines

**Fig. 5:**
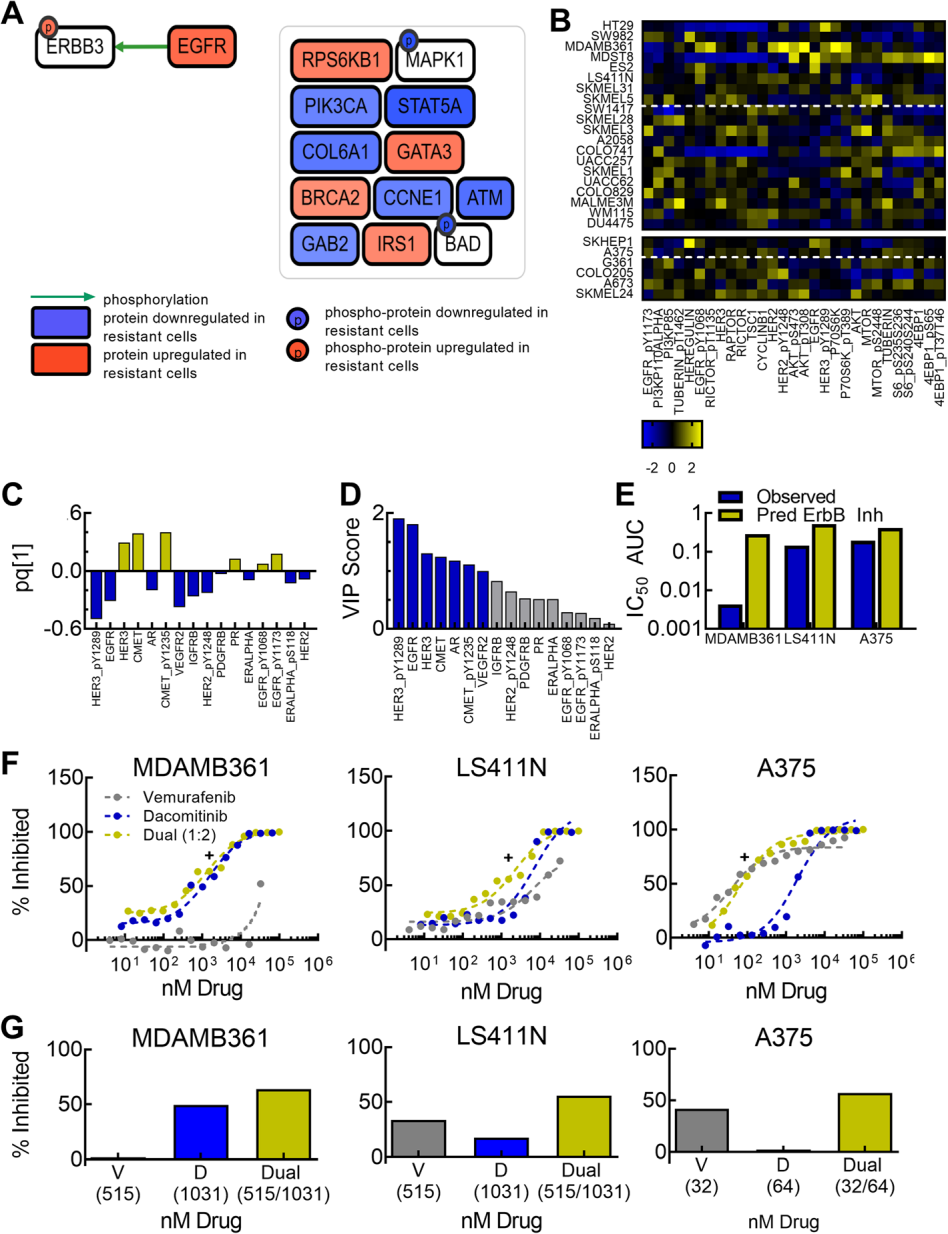
Pathway analysis of co-therapeutics to increase sensitivity to vemurafenib. (**a**) CausalPath results for protein causal relationships that are significantly up- or down-regulated in vemurafenib resistant cells (FDR = 0.2). (**b**) Heatmap of z-score normalized expression of ErbB family receptors and related downstream signaling proteins. Top heatmap indicates training set and bottom indicates testing set of cell lines in order of increasing IC_50_ AUC, with dotted line separating between AUC < 0.2. (**c**) Weights of all receptors in RPPA receptor-only O-PLS model. (**d**) VIP scores of receptors in RPPA receptor-only O-PLS model. (**e**) Comparison of IC_50_ AUC for vemurafenib monotherapy and predicted IC_50_ AUC for dual therapy with vemurafenib and a pan-ErbB inhibitor in MDA-MB-361, LS411N, and A375 cell lines. (**f**) Impact of dual pan-ErbB and BRAF inhibition using dacomitinib and vemurafenib in MDA-MB-361, LS411N, and A375 cell lines. + indicates the measured dose that was closest to the IC_50_ for dual treated. (**g**) Comparison of effects of dual treatment near the IC_50_ and the component monotherapies of vemurafenib (V) and dacotinib (D) for each cell line

Our model analysis suggested that distinct sets of proteins and phosphorylated proteins were differentially expressed among *BRAF*-V600E cell lines according to their vemurafenib sensitivity. To further analyze these proteins, we next examined their involvement in cellular signaling pathways. CausalPath is a computational method that uses biological prior knowledge to identify causal relationships that explain differential protein expression and phosphorylation [15]. Cell lines were sorted into sensitive and resistant groups based on IC_50_ AUC, and CausalPath was used to identify protein-protein interactions (PPIs) that explained significant changes in mean expression of the predictive total and phosphoproteins (VIP score > 1) in the resistant cohort of cell lines. This computational method identified that the resistant subset had increased expression of EGFR and HER3-Y1289, which could be explained by the biological prior knowledge that EGFR transphosphorylates HER3 in EGFR-HER3 heterodimers (Fig. 5a). While CausalPath identified expression patterns from PPIs, it is limited by the input proteins represented in the dataset, (i.e.*,* it cannot find the relationship A➔ B➔ C if only A and C are measured). Because the important proteins in the O-PLS model (VIP score > 1, Fig. 3c) do not include the complete cell proteome, CausalPath could not identify a full pathway, but did identify several protein interactions in the PI3K pathway, suggesting that this pathway may also be of interest (Fig. 5a). Manual curation of 29 proteins in the PI3K pathway present in the RPPA dataset are shown in a heatmap in Fig. 5b, with their projections along the principal component space of the O-PLS model in Supplemental Fig. S2. The pathway curation includes receptors, adaptor proteins, and downstream signaling cascade proteins, many of which have a VIP score greater than 1 (Additional file 9: Fig. S2A bolded). Examination of the projections of phosphorylated proteins present from this dataset shows that the majority of them project along the negative predictive component space, indicating that elevated levels correlated with more resistant cell lines (Additional file 9: Fig. S2B orange). Therefore, through CausalPath analysis and manual pathway curation, we have identified that ErbB family signaling and downstream PI3K pathway activation are upregulated in cell lines that are resistant to vemurafenib.

### Inhibition of ErbB receptors enhances sensitivity of resistant cell lines to vemurafenib

From the pathway analysis, we hypothesized that increased ErbB family signaling led to intrinsic vemurafenib resistance. As receptor-level inhibition of cellular signaling is a common therapeutic approach (e.g.*,* Herceptin), we tested whether pan-ErbB inhibition would increase vemurafenib sensitivity in the more resistant cell lines. To explore this scenario, an O-PLS model was built using the expression and activation of receptors from the RPPA dataset (16 proteins) in order to more easily simulate the impact of receptor inhibition without the confounding element of having to simulate the impact of receptor inhibition on downstream proteins. While model performance suffered (R^2^Y = 0.37, Q^2^Y = 0.12), receptors with the highest VIP scores were EGFR, HER3, and HER3 Y1289 (Fig. 5c,d). To test the hypothesis that ErbB receptor inhibition would increase vemurafenib sensitivity, inhibition was first simulated by reducing phosphorylated receptor expression in the MDA-MB-361, LS411N, A375 cell lines to that of the minimal levels detected in the data set. Vemurafenib sensitivity in these three ErbB “inhibited” cell lines was then predicted using the receptor-only O-PLS model (Fig. 5e). Simulations indicated that inhibition of ErbB pathway activity would increase sensitivity to vemurafenib across the three different tumor cell lines. To experimentally validate this prediction, we treated the MDA-MB-361, LS411N, and A375 cell lines in vitro with vemurafenib, dacomitinib (a pan-ErbB receptor tyrosine kinase inhibitor), or combination treatment of vemurafenib and dacomitinib. In comparison to either monotherapy, the IC_50_ concentrations for both drugs decreased in the combinatorial treatment, showing increased efficacy of treatment when ErbB and B-RAF were dually inhibited. Additionally, Loewe’s model values from the dose response curves indicated synergy between the two inhibitors (Fig. 5f,g, Additional file 7: Table S7). This suggests that the inhibitors worked cooperatively to target intrinsic BRAF phosphorylation (caused by the *V-600E* mutation), as well as upstream ErbB signaling that could activate pathways parallel to BRAF, including PI3K. The computational results shown here illustrate the utility of O-PLS modeling to predict vemurafenib sensitivity in an in vitro setting mimicking a basket trial. Additionally, the ease of interpreting the O-PLS model allowed for identification and in vitro validation of vulnerabilities in vemurafenib-resistant cell lines in order to increase the efficacy of treatment.

## Discussion

Using a basket trial setting of pan-cancer *BRAF*-V600E cell lines, we developed an O-PLS model to predict tumor cell sensitivity to vemurafenib and identified co-treatments to overcome inherent resistance. While others have identified signatures from transcriptomic or proteomic data that correlate to sensitivity, to attempt to expand vemurafenib use beyond *BRAF-*V600E mutations [25], the clinical reality is that the FDA-approved application of vemurafenib requires the detection of a *BRAF*-V600E mutation in advanced stage melanoma [5]. Furthermore, the drug label warns that application of vemurafenib to *BRAF* wild-type tumors can increase cell proliferation in vitro [26]. This is consistent with the move, over the past decade, to develop assays for predictive biomarkers to guide use of targeted cancer therapeutics [27]. Use of such assays, termed “companion diagnostics” [28], often increases the success rates of drugs during clinical trials [27, 29]. The approved test method and guidelines are then used for future general-population administration. Despite the failures in the non-melanoma *BRAF*-V600E basket trial for vemurafenib, the existing FDA requirement and warning for *BRAF* mutation status provide a translational structure that cannot be ignored. Through our model of protein data in pan-cancer *BRAF*-V600E cells, vemurafenib sensitivity was accurately predicted in multiple tumor cell lines including colorectal, breast, bone, and melanoma tumors. With further refinement and expansion to clinical samples, we expect that this approach could translate to refine basket trial enrollment and improve outcomes.

One of the key findings of our work is that proteomic data outperforms transcriptomic data to predict response in the basket setting. This is consistent with results obtained since the release of the RPPA expression dataset from CCLE and TCGA cohort analyses [12, 30, 31]. Their results demonstrated that in a pan-cancer model where genetic mutations are not incorporated into inclusion criteria, proteomics from RPPA outperformed RNAseq transcriptomics to predict drug sensitivity [12]. Through the outlined model comparisons shown in our study, we observed that O-PLS performed optimally when protein expression and activity were used instead of RNAseq expression. Closer analysis of individual transcript/protein/activated proteins suggests this is likely due to the disparities between protein and transcript expression or protein expression and protein activation (i.e., phosphorylation). While RPPA technology is currently used in clinical trials [32], there are situations where other protein-based assays will be needed. Chiefly, as a lysate-based measurement, RPPA from tumor biopsies will capture the protein status of the entire tumor and microenvironment, which may mask indicators of tumor cell sensitivity. As an alternative, we suggest that when RPPA is used to identify the reduced signature of highly predictive proteins in tumor cells, clinical implementation may be more accurate with techniques that enable tumor cell-specific quantification (i.e.*,* multi-spectral imaging for solid tumors, flow cytometry for non-solid tumors).

Our results also demonstrated that broad inclusion of protein expression and activity measurements can identify altered signaling pathways that influence drug response. For example, vemurafenib targets the BRAF signaling cascade and model analysis of the data supported that lines with elevated sensitivity to vemurafenib had increased phosphorylation of BRAF, MEK, and MAPK proteins (Fig. 3d bolded). While melanoma patients treated with vemurafenib have shown rapid responses to the therapy, the duration of response is often short [33], motivating a need to identify combination treatments with vemurafenib to extend progression free survival times. Results from our model suggest that melanoma cell lines initially sensitive to vemurafenib have elevated expression of p-MEK and p-BRAF when compared to inherently resistant cell lines. Recent clinical trials results showed significantly increased progression free survival and overall survival in BRAF- mutant metastatic melanomas with dual BRAF and MEK inhibitors compared to BRAF inhibitor monotherapy [34]. In constrast, the model found that cell lines with higher resistance had increased ErbB receptor-family activity and downstream PI3K signaling. Therefore, by using a method such as RPPA to expand the analysis of protein signaling beyond the targeted pathway, protein signaling activity can be better gauged and used to identify potential co-therapeutic targets in the pre-clinical setting. Additionally, through the use of models such as the O-PLS model presented here, co-treatments can be simulated to prioritize experimental testing. Specifically, we simulated dual pan-ErbB and BRAF inhibition, and validated the model prediction of a synergistic increase in sensitivity of breast, colorectal, and melanoma cell lines to vemurafenib.

While our prediction of anti-ErbB therapies was based on model analysis rather than prior knowledge, there is evidence that this synergy is clinically relevant. Our model indicated that tumor cells, including colorectal cancer cells, with increased HER3 phosphorylation exhibited increased resistance to vemurafenib. In vitro, colorectal tumor stem cells with increased HER3 expression exhibited resistance to vemurafenib in the presence of the HER3 ligand, NRG-1 [35]. Additionally, melanoma in vivo and PDX models have shown that increased ErbB family-receptor activity is associated with acquired resistance to vemurafenib [36]. While the O-PLS model presented in this study was not used to predict acquired resistance, it did identify melanoma lines with increased ErbB signaling that led to inherent vemurafenib resistance (A375). Our model and experimental results suggested that co-treatment with an ErbB inhibitor and vemurafenib would have a synergistic effect. Cetuximab, a monoclonal antibody directed towards EGFR, has been shown to increase survival in colorectal patients [37]. However, the *BRAF*-V600E colorectal patient cohort did not respond as well to cetuximab monotherapy in comparison to the wild-type *BRAF* cohort. Interestingly, in the vemurafenib basket clinical trial, colorectal patients were split into vemurafenib or vemurafenib/cetuximab treatment arm. The outcomes showed that the dual treatment arm had an increase in partial and stable responders, suggesting a potential synergy between these two inhibitors, similar to the synergy we observed in multiple tumor cell types [7].

## Conclusions

Here, we compared the predictive ability of leading machine learning algorithms for regression to predict vemurafenib sensitivity in *BRAF*-V600E cell lines from RPPA data. We determined that O-PLS predicted vemurafenib response more accurately than SVR, LASSO, and Random Forests, and the O-PLS model performed superiorly with proteomic data compared to transcriptomic data. Additionally, causal analysis identified that ErbB and PI3K signaling were upregulated in resistant cells, and that dual inhibition of ErbB receptors and BRAF increased vemurafenib sensitivity in resistant cells. Collectively, this study illustrates how an unbiased approach like O-PLS can be used to develop a model from proteomic data in a basket clinical trial setting in order to predict drug sensitivity and identify mechanisms of resistance.

## Supplementary information

**Additional file 1: Table S1.** Characterization of cell lines in training and testing sets.

**Additional file 2: Table S2.** Summary of prediction performance from regression models.

**Additional file 3: Table S3.** Binomial test results for comparison of predictive models versus O-PLS.

**Additional file 4: Table S4.** Prediction results from RNA-seq- built O-PLS models.

**Additional file 5: Table S5.** Comparison of correlation coefficients of protein/phosphoprotein/gene groups with vemurafenib IC_50_ AUC.

**Additional file 6: Table S6.** Prediction results from total protein-only and phosphoprotein-only built O-PLS models.

**Additional file 7: Table S7.** Loewes additivity model values.

**Additional file 8: Figure S1.** Prediction comparisons between O-PLS models built from total or phosphoprotein- only expression.

**Additional file 9: Figure S2.** Resistant cell lines correlate with activation of ErbB/PI3K pathway.

## Data Availability

The datasets analyzed during the current study are available in the following repositories: RPPA data was procured from the MD Anderson Cell Lines Project https://tcpaportal.org/mclp/#/ BRAF mutational status of cancer cell lines was procured through the Cancer Cell Line Encyclopedia https://portals.broadinstitute.org/ccle/data Vemurafenib sensitivity was collected as part of the Cancer Therapeutics Response Portal and normalized area-under-IC50 curve data (IC_50_ AUC) was procured from the Quantitative Analysis of Pharmacogenomics in Cancer http://tanlab.ucdenver.edu/QAPC/

## Abbreviations

IC_50_: AUC: area under IC_50_ dose response curve
LASSO: least absolute shrinkage and selection operator
O-PLS: orthogonal partial least squares
RPPA: reverse phase protein array
SVR: support vector regression
