# Supplementary material for: Tumor cell sensitivity to vemurafenib can be predicted from protein expression in a BRAF-V600E basket trial setting
Source: BMC Cancer. 2019 Oct 31;19:1025. doi: 10.1186/s12885-019-6175-2 (PMC6822426; doi:10.1186/s12885-019-6175-2)
Supplement: Supplementary file 1 — Additional file 1: Table S1. Characterization of cell lines in training and testing sets. [file 12885_2019_6175_MOESM1_ESM.docx]

**
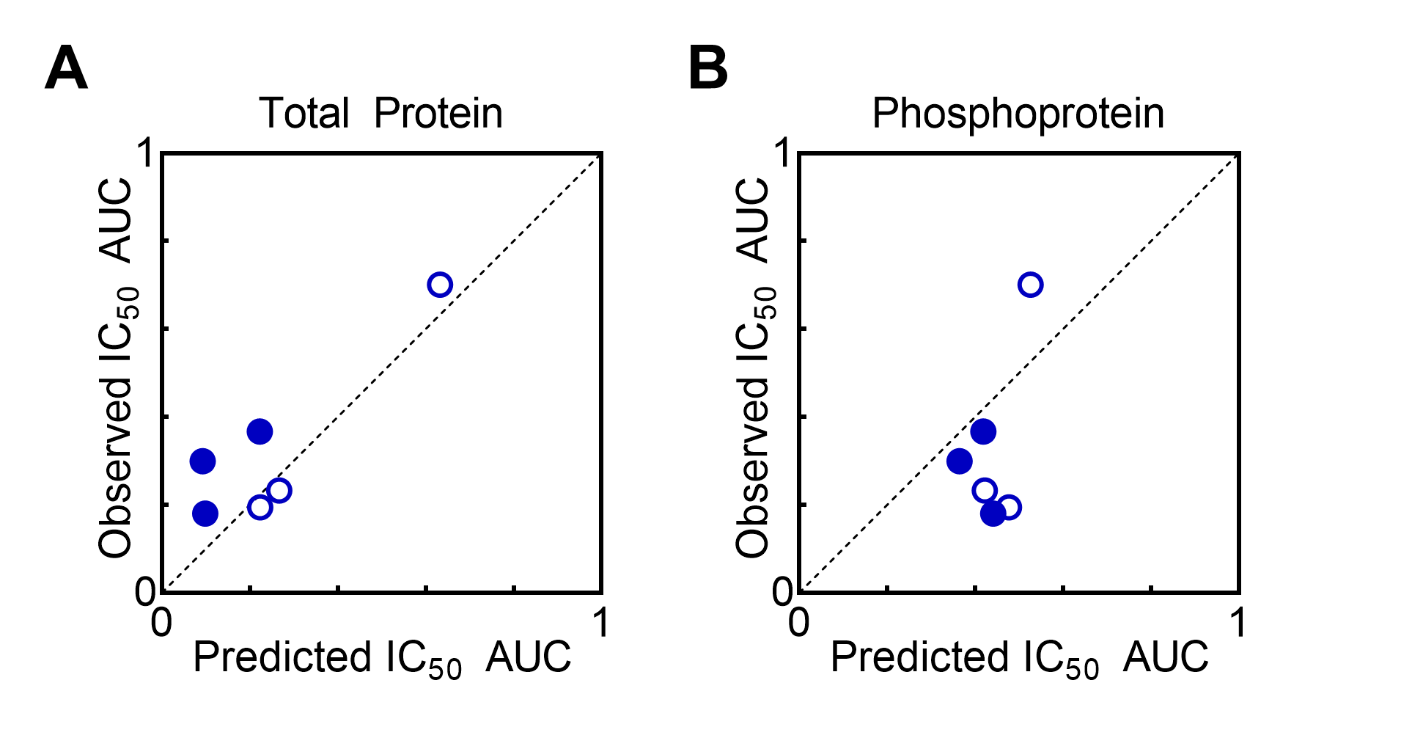
**

**Additional Figure 1. Prediction comparisons between O-PLS models built from total or phosphoprotein- only expression.** (A) O-PLS predicted IC_50_ AUC versus observed IC_50_ AUC in O-PLS model built using expression of 173 total proteins. (B) O-PLS predicted IC_50_ AUC versus observed IC_50_ AUC in O-PLS model built using expression of 59 phosphoproteins. Open symbols indicate melanoma cell lines, closed symbols indicate non-melanoma cell lines.
