# Supplementary material for: Tumor cell sensitivity to vemurafenib can be predicted from protein expression in a BRAF-V600E basket trial setting
Source: BMC Cancer. 2019 Oct 31;19:1025. doi: 10.1186/s12885-019-6175-2 (PMC6822426; doi:10.1186/s12885-019-6175-2)
Supplement: Supplementary file 2 — Additional file 2: Table S2. Summary of prediction performance from regression models. [file 12885_2019_6175_MOESM2_ESM.docx]

**
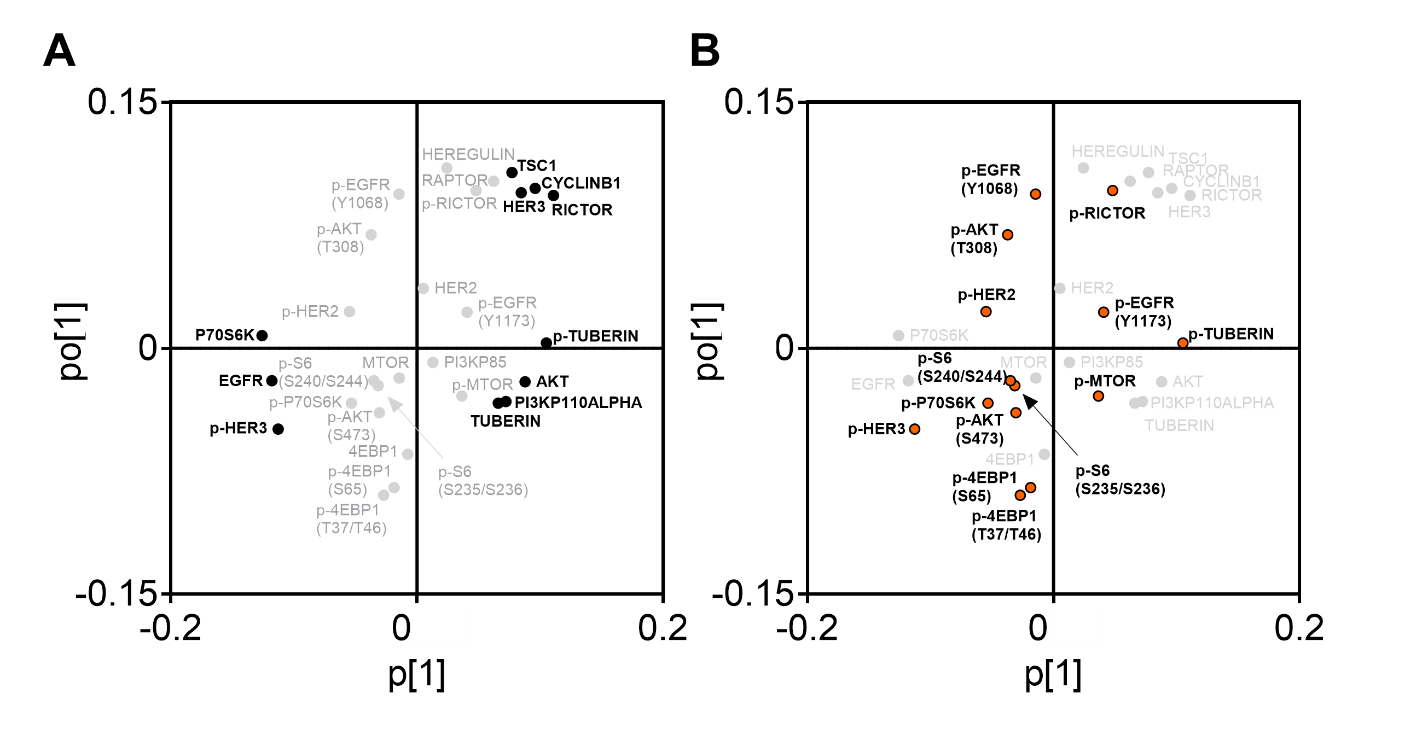
**

**Additional Figure 2. Resistant cell lines correlate with activation of ErbB/PI3K pathway**. Loadings plot of ErbB receptors and downstream PI3K pathway proteins. Proteins whose VIP scores are > 1 are bolded in (**a**) and phospho-proteins are shown bolded in (**b**).
